# Proceedings of the Clinical Microbiology Open 2022—assessing clinical laboratory and industry responses to COVID-19 pandemic testing capacity challenges (part 1)

**DOI:** 10.1128/jcm.00941-23

**Published:** 2024-02-20

**Authors:** David R. Peaper, Christopher D. Doern, Susan Sharp, On behalf of the ASM Clinical and Public Health Microbiology Committee and the ASM Corporate Council

**Affiliations:** 1 Department of Laboratory Medicine, Yale School of Medicine, New Haven, Connecticut, USA; 2 Department of Pathology, Virginia Commonwealth University Health System, Richmond, Virginia, USA; 3 Herbert Wertheim College of Medicine, Florida International University, Miami, Florida, USA; 4 Copan Diagnostics, Inc., Carlsbad, California, USA; Vanderbilt University Medical Center, Nashville, Tennessee, USA

**Keywords:** COVID-19, pandemic

## Abstract

As the COVID-19 pandemic winds down, clinical and public health laboratories, along with industry partners, reflect on the successes and failures of the pandemic response. To capture the lessons learned and better prepare for the next pandemic, the Clinical Microbiology Open (CMO) assembled key stakeholders including directors of clinical laboratories, industry partners, and state and federal agencies such as the Centers for Disease Control and Prevention and the Food and Drug Administration. Participants were asked to provide their perspectives on the initial pandemic response, supply chain constraints especially during surges, staffing challenges, test triage and communication strategies, clinical informatics needs, laboratory financial impacts of SARS-CoV-2 testing, and the emergency use authorization process. This manuscript summarizes the diagnostic laboratory and industry perspectives on these issues that were presented and discussed at CMO and proposes some steps that could be taken to improve future pandemic responses.

## INTRODUCTION

The COVID-19 pandemic strained the entire healthcare system, but among the groups, most impacted were clinical microbiology and public health laboratories as well as the biotechnology companies who supplied those laboratories with reagents and instruments. To meet the demands of the pandemic, laboratories were asked to perform testing at volumes never experienced previously (1). In addition, workforce needs increased at a time when staffing shortages and workforce turnover were already difficult issues for the laboratory (2). These factors were compounded by supply chain disruptions, a shifting regulatory landscape, and unclear guidelines further complicating laboratory operations (3).

At the same time, biotechnology companies were also facing unique demands. Processes for manufacturing scale that normally take years were expected in a period of days. Given limited quantity, difficult choices needed to be made on where to allocate scarce resources. Unfamiliarity with the emergency use authorization (EUA) process as well as changing regulatory requirements, even if reducing barriers to development, also required additional time and staff to ensure compliance.

As the pandemic wanes, we hope to reflect on how industry and the laboratory communities have met these challenges and learn from our successes and failures so that we can respond more effectively should another pandemic arise. To that end, the Clinical Microbiology Open (CMO) dedicated two separate sessions (June 2022, Washington, DC and February 2023, Carlsbad, CA) to the discussion of lessons learned throughout the pandemic. The CMO is a small format meeting dedicated to the open exchange of ideas on topics that are important to the field of diagnostic microbiology. This meeting brings together leaders in clinical and public health microbiology, industry, and federal agencies and regulatory bodies such as the Food and Drug Administration (FDA), the Centers for Disease Control and Prevention (CDC), and others. This manuscript will summarize part 1 of the two-part discussion of these issues, the first of which was held at the third Clinical Microbiology Open in Washington, DC in June of 2022.

There were 88 participants representing the diagnostics industry as part of the American Society for Microbiology (ASM) corporate council, state and federal entities, clinical laboratories from all regions of the country, and ASM leadership and staff with the bulk of attendees from clinical laboratories, including reference laboratories, and the diagnostics industry. Among diagnostics manufacturers, most areas of microbiology were represented including collection devices, molecular test systems, identification and susceptibility test systems, and rapid testing. Interested parties applied to attend the meeting, and speakers were selected based on topics outlined in applications. Not all attendees gave formal presentations, but all participated in discussions and break-out sessions. Table 1 summarizes some of the major action items and lessons learned which emerged from these discussions.

**TABLE 1:** Potential solutions discussed from lessons learned for each of the six challenge areas identified by participants of Clinical Microbiology Open

Issue	Solutions/lessons learned
Initial pandemic response	Develop a process for laboratories to pre-qualify to perform testing (e.g., FDA designated “Centers of Excellence”) with streamlined applications or pre-approval FDA and U.S. government pro-actively acquire positive specimens to facilitate test development and develop straightforward mechanism to safely distribute to ease assay development Pre-qualify equivalent sample types, collection devices, and collection media as appropriate Designate and support staffing, equipment, expertise, and logistics for regional test centers to function as “first responders” for testing
Supply chain and maintaining surge capacity	Extend expiration dates for collection and testing supplies as quickly as possible Effectively implement and rotate between hot, warm, and cold manufacturing lines Possibly learn from miliary experiences for ramping up supply chain materials and distribution mechanisms Increase communications between end users and testing suppliers, possibly through society or governmental channels Create mechanisms for individual collection sites, laboratories, or healthcare facilities to link collected specimens to excess testing capacity with minimal hurdles
Staffing	Create a coordinated clinical laboratory “crash course” curriculum for recruited non-Medical Laboratory Scientist (MLS) staff with emphasis on molecular biology from professional societies Establish relationships with traveler agencies to streamline process for surge staffing with traveling medical technologists
Test triage, communication, and allocation	Develop a system to equitably distribute testing supplies and communicate the distribution scheme with stakeholders Establish protocols within facilities to quickly triage critical testing and match testing with clinical goals Improve communication from government and industry to testing laboratories about supply chain
Informatics	Integrate tools for ordering, tracking, triaging, and resulting large volumes of test results in standard laboratory information systems Create off-the-shelf informatics tools for specimen pooling compatible with common laboratory instrumentation
Finances	Shift the financial burden for maintaining testing capacity from individual laboratories Develop alternative markets for used instrumentation to offset costs associated with scaling up and down testing through instrument acquisitions
EUA process	National societies serve as liaison between FDA to streamline the approval process Explicitly and clearly allow “bridging studies” to qualify for EUA testing Consider laboratory-developed tests without requirement for EUA Ramp up FDA staffing to manage EUA applications Minimize changes to FDA guidance documents

## LABORATORY PERSPECTIVE

Heterogeneity in laboratory structure and scope of operations among institutions necessitated different responses to the testing demands of the COVID-19 pandemic. However, multiple areas represented common challenges for laboratories, and individual laboratory responses may have varied based on geographic, temporal, and/or operational factors. The familiar challenges arising across a variety of systems are important to consider when developing laboratory responses to future pandemics.

Some health systems opted to admix high-volume, ambulatory SARS-CoV-2 testing with normal laboratory operations. Other laboratories adopted alternative approaches creating a distinct laboratory with separate space, staffing, instrumentation, etc. for testing or co-opting existing specialty laboratory space for SARS-CoV-2 testing. All approaches impacted the laboratory, staff, and operations, especially during testing surges when inpatient and ambulatory demands were the greatest. Additionally, among laboratory participants, some focused their SARS-CoV-2 testing efforts on inpatient and within-network outpatient testing, others established large-scale testing operations for specific populations (e.g., educational communities) or their surrounding communities, while others were asked to consult on the creation or expansion of alternative testing facilities.

### Initial challenges

Between March 2020 and July 2020, the primary challenge faced by laboratories was acquiring enough reagents to evaluate acutely ill patients presenting with symptoms of SARS-CoV-2 infection. For some institutions, that period represents the highest number of COVID-19-related admissions experienced during the pandemic (4). At that time, commercial assays were not widely available and, once authorized, were only available in limited quantities to most clinical laboratories. Once laboratories were able to acquire the necessary reagents and instrumentation to perform testing, they did not have sufficient clinical material to validate assays but instead had to rely upon alternative material such as *in vitro* transcripts or a difficult application process to obtain material. As a result, many laboratories had to perform less extensive validation studies in shorter time frames than was typical. Laboratories were under tremendous pressure to bring on testing quickly to support their institutions and their communities. With limited isolation room availability, the inability to rule out COVID-19 infection in persons under investigation put institutions in a difficult situation. Lastly, the limited release of testing reagents to laboratories often necessitated creative and non-standard approaches, e.g., pooling of verification data across testing sites within an institution, to test verification so as not to exhaust limited reagents with unreliable resupply schedules.

The primary testing offered early in the pandemic was through laboratory-developed tests (LDT), and expertise to run these types of assays was limited to academic medical centers and reference laboratories. While some assays became available on automated moderate- and high-complexity platforms, capacity was limited by unpredictable supply chains and strict allocation schemes that limited the availability of these tests to most laboratories even if compatible instrumentation was present. These allocation strategies were not clearly communicated to end users making it difficult to estimate a laboratory’s test capacity.

### Supply chain disruptions

Supply chain disruptions affected the laboratory in a number of ways. First, laboratories were not able to offer or perform the routine, non-COVID-19 testing. Shortages of supplies encountered during the pandemic included media for routine microbiological cultures, citrated tubes for coagulation studies, and plasma separator tubes for chemistry analysis. Additionally, there were shortages of blood supplies related to public health measures limiting gatherings and schools, an important source of blood drives, shifting to remote education.

With respect to supplies and reagents for SARS-CoV-2 testing, supply shortages negatively affected the timely testing of sick, admitted patients potentially delaying appropriate clinical and infection prevention decisions for acute care hospitals. Throughout the pandemic, some supplies might be readily available only to become scarce several months later as cases peaked internationally. Unpredictable supply chain disruptions became, especially, pronounced during the winter of 2021 to 2022 as the surge associated with the Delta variant worsened with the emergence of Omicron. These supply chain issues placed a large burden on already overwhelmed laboratories to actively manage inventory and interface with contracting and suppliers.

Early in the pandemic, supplies required for specimen collection and testing were scarce, and normal mechanisms of ordering and tracking shipments were not operational. Supplies either did not appear when expected or unexpectedly arrived alleviating an anticipated (and planned for) shortage. While many institutions have contracting or supply chain groups tasked with managing these issues, their initial focus was on obtaining necessary personal protective equipment and medical equipment (e.g., ventilators) so that care teams were able to safely care for acutely ill patients. Additionally, the lack of interchangeability of molecular reagents for testing for SARS-CoV-2 and the specificity of some orders (e.g., specific buffers or reagents) required that laboratory personnel take an active and often manual role in inventory tracking and management while simultaneously implementing new tests, training new staff, and developing disaster plans for staff in addition to their normal work responsibilities. As the pandemic progressed, some of these processes normalized, but unexpected shortages or delays continued to be a problem.

### Staffing

Many laboratories entered the COVID-19 pandemic short staffed and lacking molecular expertise. Early in the pandemic, the substantial reduction in routine clinic operations and patient avoidance of the healthcare system allowed some staff within the laboratories to be redeployed for SARS-CoV-2 testing. However, several factors contributed to continued staffing problems throughout the pandemic (5). (i) Staff were infected with SARS-CoV-2 significantly affecting operations in already lean laboratories. Depending on the stage of the pandemic, best case scenarios were absences of 6 to 14 days, but severe infections, long covid, and deaths have also affected the laboratory community. (ii) Laboratory staff were pulled back to normal duties when clinical operations resumed, leaving gaps in SARS-CoV-2 testing. In some cases, laboratory staff were expected to continue performing SARS-CoV-2 testing while also performing their regular duties contributing to burnout. (iii) Laboratories dedicated solely to COVID-19 testing rapidly expanded operations and competed for existing staff in other institutions by driving up wages. Academic medical centers and hospitals are often not well suited to quickly respond to market wage pressures leading to loss of experienced staff and reduced ability to recruit new staff. (iv) An influx of new and inexperienced staff (including those without training as medical technologists) placed tremendous training pressure on existing staff and further contributed to burnout. Less experienced staff also required greater oversight for training, education, and ongoing competency, placing an extra burden on managers and supervisors. (v) Institutions had to initiate contracts with traveler agencies to recruit and train traveling staff. Once again, the contracting, recruiting, onboarding, and training process was time-consuming for existing staff in addition to the higher institutional costs associated with large-scale use of travelers.

### Test prioritization and triaging

On an institutional level, reagent shortages necessitated the development of triage and prioritization schemes to make sure the right patients got the right test in a time frame that could facilitate decision-making. As an example of how to address the scarcity and unpredictability of testing supplies, the experience at a large academic medical center was presented at the 2022 CMO, and aspects of their experience were echoed by other attendees. They formed a health-system-wide committee to prioritize patient populations for testing. Initially, this committee met daily, and the cadence of meetings changed as the pandemic progressed. The initial task of the committee was to triage patient testing needs and communicate those priorities to the entire health system. To facilitate communication, the committee described testing as a 2-h test, 6-h test, or >24-h test, and this framework endured throughout the pandemic. Patient populations (e.g., symptomatic, admitted patients) were then prioritized for testing on different test systems to meet critical turn-around times (TATs).

Patient population and acuity were determined by Ask at Order Entry Questions within the electronic medical record. Symptom status as well as basic underlying clinical information (e.g., urgent procedure, patient in labor, and patient to intensive care unit) was captured. This information was used to drive unique icons on specimen labels to inform the laboratory which testing platform should be used. The laboratory used the icons in conjunction with delivery mechanism (e.g., hand delivery vs courier) to select the appropriate test system. Among all participants, supply chain challenges meant that laboratories implemented redundant COVID-19 test options to ensure that when one supply chain failed, backup tests were available. As a result, it was common for laboratories to have four or more testing platforms for detecting SARS-CoV-2, and institutions and laboratories were forced to develop their own solutions to these problems.

Many laboratories used a hybrid approach with testing some specimens locally while sending other specimens to health system or regional or national reference laboratories. Long turn-around times were seen for reference testing at different points in the pandemic, but TAT improved as testing capacity increased and patterns of testing stabilized. Extended TATs were again seen in the winter of 2021 to 2022 with the international Delta and Omicron surges. Regardless of TAT, sending specimens out was an important “relief valve” for some institutions, allowing them to focus on testing acutely ill patients under their institutional umbrellas. However, reference testing typically requires specific contractual, logistical, and informatics arrangements, and establishing reference contracts with newly created specialized SARS-CoV-2 testing laboratories may not have been undertaken in favor of existing reference laboratory relationships. This may have led to the underutilization of collective testing capacity in times of need.

### Informatics

Informatics challenges existed throughout the pre-analytical, analytical, and post-analytical phases of testing. From a pre-analytical perspective, ordering, tracking, and receiving large volumes of specimens by the laboratory created unique informatics needs. While reference laboratories have a robust infrastructure to handle such volumes, hospital-based laboratories had to take on high-volume testing requiring optimization of existing laboratory information systems. Alternatively, novel front-end solutions were developed in some cases. The widespread acceptance of specimen pooling allowed reagents to be extended, but there were no off-the-shelf informatics solutions to automate the creation and deconvolution of pool information while preserving specimen identifiers throughout the process. Laboratories who were performing pooling needed to develop novel approaches to solve these problems including manual methods or custom informatics solutions.

An underappreciated barrier laboratories faced was effectively communicating testing results directly to patients. Traditionally, laboratory testing is ordered by a provider, and laboratories would report results to providers who would then communicate results to patients. Prior to the COVID-19 pandemic, direct patient communication mechanisms were developed (e.g., MyChart), but the provider was still central to the ordering, resulting, and follow-up processes. The advent of mass-testing strategies for SARS-CoV-2 eliminated providers from the ordering and resulting process for a considerable number of patients and tests. Testing laboratories had to rely upon existing reporting tools or develop new tools to communicate results, but many relied upon email, text messaging, or dedicated web portals requiring pre-registration, correct contact information, or internet access creating concerns about equity and access to testing. The importance of timely and effectively linking patients to care was emphasized with the development of anti-SARS-CoV-2 therapies, including monoclonal antibodies and oral medications. Some healthcare institutions set up or repurposed clinical call centers to connect eligible patients to therapy.

### Finances

SARS-CoV-2 placed tremendous financial strains on healthcare institutions (6). Large-scale COVID-19 testing of ambulatory populations could be financially positive for laboratories, but these funds could be institutionalized so that laboratories may not financially benefit from testing. Pricing for reference lab testing often matched or exceeded public health reimbursement, potentially costing hospitals and laboratories more money. However, some laboratories may be distinct business entities with positive revenues that are directly re-invested in the laboratory. In these cases, large-scale SARS-CoV-2 testing may have been viewed more favorably despite the struggles associated with this testing.

A number of laboratories made substantial investments in new instrumentation during the COVID-19 pandemic. In some cases, this may have accelerated acquisition of new instrumentation, but, in many cases, multiple testing platforms were acquired that would be unutilized or underutilized outside of surge conditions. There is not a straightforward process to cease testing on these instruments while maintaining reagents and competency within laboratories without cost. The acquisition and maintenance of these instruments also come at a cost that may undermine non-pandemic-related efforts at standardization and consolidation that may reduce overall cost and improve operations. Finally, as COVID testing wound down, stockpiled supplies and reagents expired, forcing laboratories and hospitals to absorb these costs.

As discussed above, staffing was a major issue for laboratories. For laboratories that added staff specifically for SARS-CoV-2 testing, fluctuating testing volume presented challenges for maintaining staffing levels. Laboratories do not typically keep “reserve staff” in case of future needs, but this may leave laboratories short staffed with reduced testing capabilities in the case of future surges. The skill set developed for molecular microbiology testing may not directly transfer to other areas of the laboratory, and staff may have been lost to attrition or lay-offs creating gaps during subsequent surges.

### Emergency use authorization process

The SARS-CoV-2 pandemic represented the first time most clinical laboratories had to rely upon tests available through EUA (7). The early requirement for EUA submission for all tests for SARS-CoV-2 delayed the development and implementation of some tests by clinical laboratories due to a lack of experience or expertise with the FDA submission process. However, once it became clear that commercially available tests would not be able to meet the testing demands, numerous laboratories submitted applications for EUA of LDTs or modified FDA-authorized tests. However, obtaining appropriate validation material was not easy, potentially adversely affecting EUA applications. The FDA-provided molecular template was helpful, and, in most cases, direct feedback from the FDA prior to submission was timely and responsive. Initially, there was the ability to perform “bridging studies” allowing for test modifications to be internally validated. There was also a period where the FDA considered the existing LDT framework sufficient for SARS-CoV-2 testing without a requirement for EUA submission from individual laboratories. However, guidance regarding bridging studies, EUA submission, and LDT applicability changed over time, and review and feedback for individual EUA submissions became prolonged with uncertainty around prioritization and review schedule. Furthermore, as guidance documents changed, submissions were held to standards that were not in place originally at the FDA.

### Laboratory action items

Clinical microbiology laboratories are not accustomed to keeping a “reserve” of instruments, supplies, and staff to meet the need of a pandemic, and there is not a mechanism, other than institutional funds, to maintain these resources. Federal, state, and/or local resources and support would be needed to maintain this reserve capacity without undermining institutional finances. While this may not be applicable for all sites, pathways could be created for institutions to receive support in exchange for expectations around testing ramp-up and capacity. A grant program analogous to the regional emerging pathogens model could be used.

The allocation of reagents and instruments was not transparent, and many laboratories and hospitals were on their own to obtain needed resources. Clinical laboratories had to rely upon manual, highly complex assays, while some clinical studies and reference laboratories received moderately complex or automated assays. Additionally, laboratories dealt with uncertainty around when instruments and reagents would arrive which encouraged redundant or duplicative instrument acquisitions and stockpiling. Industry support for a more robust market for used instrumentation would facilitate the redistribution of instruments among laboratories to help streamline testing.

Laboratory staffing continues to be a major concern, and it represents a chronic problem at many institutions. A streamlined process for finding, onboarding, and training staff is needed. Educational resources are needed to help fulfill background and training requirements for new staff, who often have less experience, in molecular principles and methods. The American Society for Microbiology is developing such a curriculum, and collaboration with other groups such as the American Society of Clinical Pathology, the College of American Pathologists, and the Association for Public Health Laboratories will further facilitate the availability of this information.

For laboratories performing LDTs who could potentially pursue an EUA, uncertainty around reagents, test systems, and specimen types that might qualify for testing should be clarified. The CDC assay was initially authorized with a limited number of extraction and amplification platforms, but that list grew substantially. “Pre-qualification” of platforms with representative pathogens could reduce time to implementation. Additionally, equivalent sample types could be pre-qualified for appropriate use from an analytical perspective.

## INDUSTRY PERSPECTIVE

During the 2022 Clin Micro Open, industry representatives from several companies served on a panel to discuss their experiences mitigating the global shortages of supplies, staffing issues, and regulatory issues. Below are highlights from the companies’ experiences during the pandemic-managing issues such as surge capacity, shortage of raw materials, and staffing. To avoid the perception of bias, manufacturers have been anonymized.

### Manufacturer experiences

Manufacturer A produces specimen collection devices for diagnostic testing. At the height of the pandemic, the company’s main production facilities in Europe changed from a normal two shifts/5 days a week to 24-7 production. To address the worldwide swab/media shortages in the United States, swabs were shipped by military transport from Europe in collaboration with the U.S. government. This manufacturer doubled its manufacturing capacity during the pandemic and established facilities for increased production, new storage facilities, and a new technology center to oversee automation services. As demand continued to rise globally, the company opened a new production facility in California and worked with Apple (Cupertino, CA) through a special grant to set up additional production lines for swabs and media. An additional plant in Puerto Rico likewise ramped up for swab and media production. To help meet demand, manufacturer A granted a limited patent licensing program so that other companies could manufacture and supply flocked swabs. They also worked with the Food and Drug Administration to assist in the authorization of the use of Amies media and several different swab types to help meet the demand for collection devices.

Manufacturer B is a global medical technology company that manufactures syringes, infusion pumps, as well as other equipment and is a large supplier of tubing globally. Manufacturer B offered a perspective on the impacts of the SARS-CoV-2 pandemic on device manufacturing. Early in the pandemic, manufacturer B received requests for longer-than-standard tubing to accommodate greater physical separation between hospitalized COVID-19 patients and other patients. In addition to manufacturing in the U.S., the company has manufacturing facilities in Asia. This international process for receiving products in the U.S. was disrupted when borders were closed, and manufacturer B also opened additional manufacturing facilities in the U.S. in response. In addition, the reliance on semi-conductor chips to deliver connected care for patients and the shortages of material to produce these chips also contributed to manufacturing issues. This challenge affected many manufacturers in the medical device industry.

Manufacturer C produces molecular diagnostic platforms; they have manufacturing facilities in Europe and in the U.S. The company learned of the developing pandemic in January of 2020, during a global sales meeting that included representatives from Wuhan, China. As the pandemic developed, the company’s production increased significantly during 2020 to 2021, doubling its production of molecular testing devices in 2020 and doubling production again in 2021. The company kept “cold” (non-operating) production lines with federal support; maintained “warm” (operating at low capacity) lines, which cycle in and out; and ran “hot” (operating at full capacity) lines. Manufacturer C reported being instructed by the U.S. government that it could no longer export its product outside of the country. The company also experienced staffing shortages for technicians to service instruments in hospitals and faced personnel oversight challenges when supervisory staff went to remote working, while manufacturing staff were still on-site.

Manufacturer D is a small start-up plant in California. This company worked with the FDA early in the pandemic to create an assay for SARS-CoV-2 detection and genotyping, and soon many public health laboratories were using their test. The company used an approach that evaluated maximum flexibility vs maximum efficiency in instrument operation. During the pandemic, it learned how to react quickly to a changing environment. Though detection test capacity was a key need early in the pandemic, as new variants of concern appeared, whole-genome sequencing emerged as a key public health need, and usage of their instruments shifted toward surveillance rather than detection. The high complexity of whole-genome sequencing assays requires even greater staffing requirements than detection assays, and by fully automating the whole-genome sequencing process, the company enabled many more laboratories to perform surveillance testing.

The key lessons learned from industry experiences centered around staffing, supply issues, and communications. The following discussion will focus on some of the action items that can be taken to capitalize on the industry lessons which were learned.

### Industry action items

Industry representatives from all areas of diagnostic microbiology participated in the 2022 CMO discussion regarding lessons learned from the COVID-19 pandemic. The panelists, as well as audience participants, elaborated on the challenges they faced throughout, and, despite the differences in the products they provide, there were some common themes and lessons that could be learned. Summarized below are a few of the key take-home points along with some action items which may help us better respond to the next pandemic.

#### Staffing

Industry representatives all agreed that there is an increased workload associated with the FDA EUA regulatory process, and companies required additional regulatory staff to manage these submissions. During the discussion, it was suggested that ASM might be able to help with the regulatory process by serving as a liaison between FDA and ASM’s industry colleagues to streamline the approval process.

#### Supply chain and surge capacity

Another need expressed by industry was communicating to the federal government a short list of components that need to be reserved for managing production of healthcare supplies during public health emergencies. During the pandemic, industry could not reliably procure raw materials, such as aluminum, plastics, etc., needed to produce and package their products. The material shortages were partly a result of needing to compete with other manufacturers for these limited supplies. Industry now has an even greater sensitivity regarding access to raw materials and the need to diversify suppliers. The industry representatives indicated that they have learned that secondary and tertiary suppliers are necessary and that you may need to go to manufacturers outside of the U.S., understanding that this strategy may also be associated with increasingly complex shipping issues.

Regarding the maintenance of manufacturing surge capacity, it was discussed that companies are in a better place today than they were in 2019 and should not face the same issues should the need to surge production rise again. The federal government is one of the largest, if not the largest, consumers for many products, but there is a lack of transparency regarding what the government does with these products. Greater transparency could help with the understanding of distribution of supplies by the government. Staffing challenges in industry included dealing with waves of ramping up production of supplies and needing extra personnel, laying people off between COVID-19 waves, and then difficulty ramping up again as cases once again surged. These experiences will help companies better meet staffing needs going forward. The importance of keeping production lines “warm” while continuing to innovate with new products is important for continued success and viability in the industrial realm.

To maintain manufacturing surge testing capacity, several steps can be taken, including FDA extension of expiration dates for collection devices and other materials. This is already being done by the FDA to some extent with testing materials. It was felt that some companies might benefit from military expertise on how to handle ramping up and down of supplies. Industry needs to better understand the needs of individual end users, including both the diagnostic testing laboratories as well as those in pharmaceutical clinical studies, which rely on a supply of collection devices and testing platforms for vaccine and therapy studies. As already mentioned, enhanced communication between laboratories and industry can assist in the understanding of each other’s needs/positions and help to build appropriate reserves.

#### Communication and allocation

The need for communication about the availability of industry products was critical during the pandemic. There have been many misunderstandings about supply issues. As an example, there was a misperception that there was a shortage of collection swabs in Europe. Though this was not the reality, manufacturer A was criticized for sending swabs out of the country. Additionally, in the U.S., there was little understanding of what industry was doing to help combat the pandemic. Industry representatives reached out to ASM and asked for their assistance with communications to diagnostic laboratories about what was being done to ramp up production of testing materials. This type of communication can be key in helping both entities mitigate the challenges of the pandemic.

During the pandemic, a substantial portion of collection devices and SARS-CoV-2 testing kits were supplied to the federal government. Greater transparency and communication by the federal government on its use and dispersion of these supplies are important going forward. Industry also needs to pay close attention to stockpiling, and how to handle the production and distribution of products for patient care vs vaccine trials, large reference laboratories vs hospital laboratories, etc.

#### EUA process

Regarding the EUA process, from the industry perspective, several issues were brought forward. There needs to be an increased understanding of the EUA process. It was suggested that an *ad hoc* group of industry partners could meet with the FDA and that the FDA might consider establishing a tiered system for different types of pandemics and timelines for testing priorities that can be shared with industry. Another suggestion was that perhaps some laboratories could be FDA “centers of excellence” and work with industry to rapidly bring up EUAs. In addition, it was discussed that it would be a great advantage to have a system in place with the FDA and the U.S. government to acquire positive specimens from around the world to share them as soon as possible for EUA test development.

## LIMITATIONS

In this manuscript, we sought to provide a summary of the presentations and discussions that occurred among attendees at the third CMO meeting. While there were representatives from multiple clinical laboratories, diagnostic companies, and federal agencies, the perspectives presented were limited to those in attendance, and the full spectrum of testing laboratories and manufacturers were not represented. Additionally, the nature of the talks presented and discussion dealt heavily with individual company or institutional challenges and responses, and there was not a systematic review of data. Thus, it is impossible to quantify the exact issues that were raised, but we sought to present the recurring discussion points that were presented at the meeting. Furthermore, laboratories, the diagnostic industry, and state and federal agencies continued to respond and evolve practices and guidance since the time of this meeting. Finally, the attendees of this meeting are not empowered to act upon many of the identified action items, but the identified items and framework may be used by ASM and other entities to advocate for changes to the approach to future pandemics.

## SUMMARY

In general, discussants were positive about the pandemic response and how challenges were addressed, given the circumstances that existed at the time. A unifying concern centered around maintaining the capacity to surge testing and manufacturing of supplies. However, it was felt that laboratories and industry are in a much better position to meet those needs than prior to the pandemic. In addition, there is still room for improvement in the EUA process, and it was felt that some clarifications are still needed. Furthermore, it was suggested that it may be useful to allow laboratories to pre-qualify for approval to perform EUA testing which would expedite the process when needed. Another commonly held opinion was that communication and transparency are critical for all parties involved in a pandemic response. One of the biggest challenges the diagnostic community faced was the lack of a system to equitably distribute testing supplies. Throughout the pandemic, there were significant questions around the supply chain shortages and a lack of transparency in the allocation process.

Finally, it was concluded that industry and clinical microbiology successfully managed the pandemic’s many challenges. Mistakes were made, and there is room for improvement, but we are all better positioned to address future pandemics because of the lessons which have been learned.
